# (*E*)-1-(2,4-Dinitro­phen­yl)-2-[1-(3-nitro­phen­yl)ethyl­idene]hydrazine

**DOI:** 10.1107/S1600536812034812

**Published:** 2012-08-11

**Authors:** Hoong-Kun Fun, Suchada Chantrapromma, Boonlerd Nilwanna, Thawanrat Kobkeatthawin, Nawong Boonnak

**Affiliations:** a X-ray Crystallography Unit, School of Physics, Universiti Sains Malaysia, 11800 USM, Penang, Malaysia; b Crystal Materials Research Unit, Department of Chemistry, Faculty of Science, Prince of Songkla University, Hat-Yai, Songkhla 90112, Thailand

## Abstract

In the asymmetric unit of the title compound, C_14_H_11_N_5_O_6_, there are three crystallographically independent mol­ecules with similar conformations but some differences in bond angles. The mol­ecules are slightly twisted with the dihedral angles between the benzene rings being 10.02 (14), 8.41 (15) and 1.40 (14)°. In each mol­ecule, an intra­molecular N—H⋯O hydrogen bond generates an *S*(6) ring motif. In the crystal, mol­ecules are linked by weak C—H⋯O inter­actions into a three-dimensional network. π–π inter­actions with centroid–centroid distances of 3.5635 (17)–3.8273 (18) Å are observed.

## Related literature

For bond-length data, see: Allen *et al.* (1987 ▶). For hydrogen-bond motifs, see: Bernstein *et al.* (1995 ▶). For a related structure, see: Chantrapromma *et al.* (2011 ▶). For background to and the biological activity of hydro­zones, see: Cui *et al.* (2010 ▶); Krishnamoorthy *et al.* (2011 ▶); Molyneux (2004 ▶); Raja *et al.* (2012 ▶); Sathyadevi *et al.* (2012 ▶). For the stability of the temperature controller used in the data collection, see: Cosier & Glazer (1986 ▶).
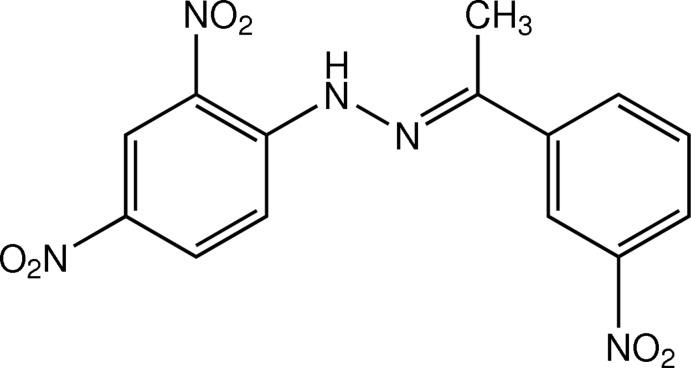


## Experimental

### 

#### Crystal data

C_14_H_11_N_5_O_6_
*M* *_r_* = 345.28
Monoclinic, 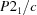

*a* = 7.3309 (2) Å
*b* = 38.3569 (8) Å
*c* = 16.8027 (4) Å
β = 115.158 (1)°
*V* = 4276.57 (18) Å^3^
*Z* = 12
Mo *K*α radiation
μ = 0.13 mm^−1^
*T* = 100 K
0.49 × 0.10 × 0.10 mm

#### Data collection

Bruker APEXII CCD area-detector diffractometer
Absorption correction: multi-scan (*SADABS*; Bruker, 2005 ▶) *T*_min_ = 0.940, *T*_max_ = 0.988
49752 measured reflections
12460 independent reflections
7719 reflections with *I* > 2σ(*I*)
*R*_int_ = 0.072

#### Refinement

*R*[*F* ^2^ > 2σ(*F* ^2^)] = 0.077
*wR*(*F* ^2^) = 0.159
*S* = 1.07
12460 reflections
679 parameters
H-atom parameters constrained
Δρ_max_ = 0.51 e Å^−3^
Δρ_min_ = −0.38 e Å^−3^

### 

Data collection: *APEX2* (Bruker, 2005 ▶); cell refinement: *SAINT* (Bruker, 2005 ▶); data reduction: *SAINT*; program(s) used to solve structure: *SHELXTL* (Sheldrick, 2008 ▶); program(s) used to refine structure: *SHELXTL*; molecular graphics: *SHELXTL*; software used to prepare material for publication: *SHELXTL* and *PLATON* (Spek, 2009 ▶).

## Supplementary Material

Crystal structure: contains datablock(s) global, I. DOI: 10.1107/S1600536812034812/is5180sup1.cif

Structure factors: contains datablock(s) I. DOI: 10.1107/S1600536812034812/is5180Isup2.hkl

Supplementary material file. DOI: 10.1107/S1600536812034812/is5180Isup3.cml

Additional supplementary materials:  crystallographic information; 3D view; checkCIF report

## Figures and Tables

**Table 1:** Hydrogen-bond geometry (Å, °)

*D*—H⋯*A*	*D*—H	H⋯*A*	*D*⋯*A*	*D*—H⋯*A*
N1*A*—H1*AA*⋯O1*A*	0.88	1.98	2.615 (3)	128
N1*B*—H1*BA*⋯O1*B*	0.88	2.01	2.637 (3)	127
N1*C*—H1*CA*⋯O1*C*	0.88	1.98	2.617 (3)	129
C3*A*—H3*AA*⋯O2*A* ^i^	0.95	2.44	3.320 (3)	154
C3*B*—H3*BA*⋯O2*C* ^ii^	0.95	2.40	3.188 (3)	140
C3*C*—H3*CA*⋯O2*B* ^iii^	0.95	2.52	3.413 (3)	156
C14*B*—H14*F*⋯O4*A* ^iv^	0.98	2.53	3.316 (4)	137
C14*C*—H14*J*⋯O4*C* ^iii^	0.98	2.54	3.253 (4)	129

Symmetry codes: (i) 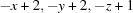; (ii) 
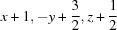
; (iii) 
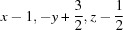
; (iv) 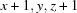.

**Fig. 1.**
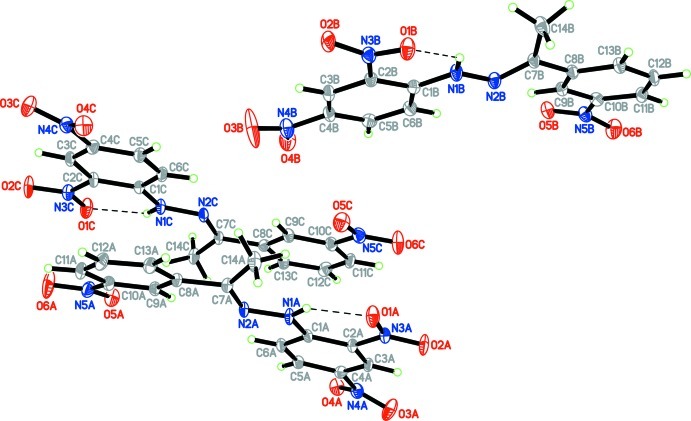
The asymmetric unit of the title compound, showing 40% probability displacement ellipsoids and the atom-numbering scheme. Hydrogen bond is shown as a dashed line.

**Fig. 2.**
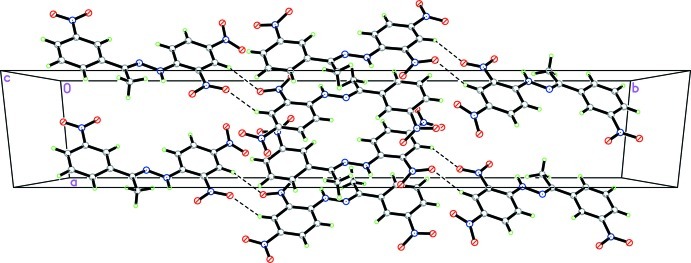
The crystal packing diagram of the title compound viewed approximately along the c axis. Hydrogen bonds are shown as dashed lines.

## supplementary crystallographic information

### Comment

Considerable attentions have been devoted to hydrazones and their complexes which have been acknowledged to possess diverse biological properties as antibacterial, antifungal, anagesic, anti-inflammatory as well as antioxidant activities (Cui *et al.*, 2010; Krishnamoorthy *et al.*, 2011; Raja *et al.*, 2012; Sathyadevi *et al.*, 2012). In our on-going research on bioactivity of hydrazones, the title compound (I) was synthesized and evaluated for antioxidant activity by DPPH scavenging (Molyneux, 2004) and was found to be weakly active with 30% inhibition. It was also screened for antibacterial activity and found to be inactive. Herein we report the synthesis and crystal structure of (I).

In the asymmetric unit of (I), C_14_H_11_N_5_O_6_, there are three crystallographically independent molecules *A*, *B* and *C* with similar conformations but some differences in bond angles (Fig. 1). The molecular structure is slightly twisted with the dihedral angle between the two benzene rings being 10.02 (14), 8.41 (15) and 1.40 (14)° in molecule *A*, *B* and *C*, respectively. The central ethylidenehydrazine bridge is planar with the torsion angles N1–N2–C7–C14 = 2.6 (4), -3.1 (4) and -2.1 (3)° in molecules *A*, *B* and *C*, respectively. This central N1/N2/C7/C14 plane makes dihedral angles of 12.90 (18) and 20.11 (18)° with the 2,4-dinitro- and 3-nitro-substituted benzene rings, respectively in molecule *A*, whereas the corresponding values are 12.50 (18) and 20.51 (18)° in molecule *B*, and 11.70 (18) and 12.33 (18)° in molecule *C*. In all three molecules, the nitro group of 3-nitrophenyl are co-planar with their bound benzene rings with *r.m.s*. deviations of 0.079 (2), 0.030 (2) and 0.025 (3) Å in molecules *A*, *B* and *C*, respectively, for the nine non H-atoms (C8–C13/N5/O5–O6). In the 2,4-dinitrophenyl, the *ortho*-nitro group lies on the same plane with its bound benzene ring with *r.m.s*. deviations of 0.023 (2), 0.075 (2) and 0.025 (2) Å in molecules *A*, *B* and *C*, repectively, for the nine non H-atoms (C1–C6/N3/O1–O2), but the *para*-nitro group is deviated with the torsion angles O3–N4–C4–C3 = 19.8 (3)° and O4–N4–C4–C3 = -160.9 (2)° in molecule *A* whereas the corresponding values are -9.8 (4) and 170.6 (2)° in molecule *B*, and -21.0 (3) and 159.4 (2)° in molecule *C*. In each molecule, intramolecular N—H···O hydrogen bonds between the hydrazone-NH and the *ortho*-nitro group (Fig. 1 and Table 1) generate three S(6) ring motifs (Bernstein *et al.*, 1995). The bond distances are in normal ranges (Allen *et al.*, 1987) and are comparable with the related structure (Chantrapromma *et al.*, 2011).

In the crystal structure (Fig. 2), the molecules are linked by C—H···O weak interactions (Table 1) into a three-dimensional network. π–π interactions were presented with the distances of *Cg*1···*Cg*6 = 3.5635 (17) Å, *Cg*2···*Cg*5 = 3.5650 (18) Å, *Cg*3···*Cg*4^v^ = 3.6544 (18) Å and *Cg*3···*Cg*4^vi^ = 3.8273 (18) Å [symmetry codes: (v) 1 - *x*, 2 - *y*, 2 - *z*; (vi) 2 - *x*, 2 - *y*, 2 - *z*]; *Cg*1, *Cg*2, *Cg*3, *Cg*4, *Cg*5 and *Cg*6 are the centroids of C1A–C6A, C8A–C13A, C1B–C6B, C8B–C13BA, C1C–C6C and C8C–C13C benzene rings, respectively.

### Experimental

The title compound (I) was synthesized by dissolving 2,4-dinitrophenylhydrazine (0.40 g, 2 mmol) in ethanol (10.00 ml) and H_2_SO_4_ (conc.) (98%, 0.50 ml) was slowly added with stirring. 3-Nitroacetophenone (0.35 g, 2 mmol) in ethanol (10.00 ml) was then added to the solution with continuous stirring. The solution was refluxed for 1 hr yielding an yellow solid which was filtered off and washed with methanol. Yellow block-shaped single crystals of the title compound suitable for *X*-ray structure determination were recrystalized from ethanol by slow evaporation of the solvent at room temperature over several days (m.p. 507–508 K).

### Refinement

All H atoms were positioned geometrically and allowed to ride on their parent atoms, with N—H = 0.88 Å, C—H = 0.95 Å for aromatic and 0.98 Å for CH_3_ atoms. The *U*_iso_(H) values were constrained to be 1.5*U*_eq_ of the carrier atom for methyl H atoms and 1.2*U*_eq_ for the remaining H atoms. A rotating group model was used for the methyl groups.

### Crystal data

**Table:**

C_14_H_11_N_5_O_6_	*F*(000) = 2136
*M**_r_* = 345.28	*D*_x_ = 1.609 Mg m^−^^3^
Monoclinic, *P*2_1_/*c*	Melting point = 507–508 K
Hall symbol: -P 2ybc	Mo *K*α radiation, λ = 0.71073 Å
*a* = 7.3309 (2) Å	Cell parameters from 12460 reflections
*b* = 38.3569 (8) Å	θ = 1.1–30.0°
*c* = 16.8027 (4) Å	µ = 0.13 mm^−^^1^
β = 115.158 (1)°	*T* = 100 K
*V* = 4276.57 (18) Å^3^	Block, yellow
*Z* = 12	0.49 × 0.10 × 0.10 mm

### Data collection

**Table:**

Bruker APEXII CCD area-detector diffractometer	12460 independent reflections
Radiation source: sealed tube	7719 reflections with *I* > 2σ(*I*)
Graphite monochromator	*R*_int_ = 0.072
φ and ω scans	θ_max_ = 30.0°, θ_min_ = 1.1°
Absorption correction: multi-scan (*SADABS*; Bruker, 2005)	*h* = −10→10
*T*_min_ = 0.940, *T*_max_ = 0.988	*k* = −49→53
49752 measured reflections	*l* = −22→23

### Refinement

**Table:**

Refinement on *F*^2^	Primary atom site location: structure-invariant direct methods
Least-squares matrix: full	Secondary atom site location: difference Fourier map
*R*[*F*^2^ > 2σ(*F*^2^)] = 0.077	Hydrogen site location: inferred from neighbouring sites
*wR*(*F*^2^) = 0.159	H-atom parameters constrained
*S* = 1.07	*w* = 1/[σ^2^(*F*_o_^2^) + (0.0437*P*)^2^ + 3.8937*P*] where *P* = (*F*_o_^2^ + 2*F*_c_^2^)/3
12460 reflections	(Δ/σ)_max_ = 0.001
679 parameters	Δρ_max_ = 0.51 e Å^−^^3^
0 restraints	Δρ_min_ = −0.38 e Å^−^^3^

### Special details

**Table:**

Experimental. The crystal was placed in the cold stream of an Oxford Cryosystems Cobra open-flow nitrogen cryostat (Cosier & Glazer, 1986) operating at 120.0 (1) K.
Geometry. All e.s.d.'s (except the e.s.d. in the dihedral angle between two l.s. planes) are estimated using the full covariance matrix. The cell e.s.d.'s are taken into account individually in the estimation of e.s.d.'s in distances, angles and torsion angles; correlations between e.s.d.'s in cell parameters are only used when they are defined by crystal symmetry. An approximate (isotropic) treatment of cell e.s.d.'s is used for estimating e.s.d.'s involving l.s. planes.
Refinement. Refinement of *F*^2^ against ALL reflections. The weighted *R*-factor *wR* and goodness of fit *S* are based on *F*^2^, conventional *R*-factors *R* are based on *F*, with *F* set to zero for negative *F*^2^. The threshold expression of *F*^2^ > σ(*F*^2^) is used only for calculating *R*-factors(gt) *etc*. and is not relevant to the choice of reflections for refinement. *R*-factors based on *F*^2^ are statistically about twice as large as those based on *F*, and *R*- factors based on ALL data will be even larger.

### Fractional atomic coordinates and isotropic or equivalent isotropic displacement parameters (Å^2^)

**Table:**

	*x*	*y*	*z*	*U*_iso_*/*U*_eq_	
O1A	1.0005 (3)	0.92844 (5)	0.64127 (11)	0.0268 (4)	
O2A	0.9875 (3)	0.97771 (5)	0.57674 (12)	0.0276 (4)	
O3A	0.6139 (3)	0.99217 (5)	0.26790 (13)	0.0359 (5)	
O4A	0.3472 (3)	0.95996 (5)	0.20354 (11)	0.0254 (4)	
O5A	0.2251 (3)	0.75669 (5)	0.35614 (12)	0.0261 (4)	
O6A	0.2531 (4)	0.70272 (5)	0.39490 (14)	0.0472 (6)	
N1A	0.7915 (3)	0.87484 (5)	0.55324 (13)	0.0172 (4)	
H1AA	0.8764	0.8817	0.6060	0.021*	
N2A	0.7233 (3)	0.84100 (5)	0.53897 (13)	0.0171 (4)	
N3A	0.9363 (3)	0.94709 (5)	0.57465 (13)	0.0177 (4)	
N4A	0.5092 (3)	0.96680 (6)	0.26601 (14)	0.0209 (5)	
N5A	0.3093 (3)	0.73305 (6)	0.40790 (14)	0.0241 (5)	
C1A	0.7255 (4)	0.89752 (6)	0.48449 (15)	0.0151 (5)	
C2A	0.7932 (3)	0.93258 (6)	0.49190 (15)	0.0150 (5)	
C3A	0.7244 (4)	0.95503 (6)	0.41999 (16)	0.0167 (5)	
H3AA	0.7756	0.9781	0.4254	0.020*	
C4A	0.5813 (4)	0.94319 (6)	0.34122 (15)	0.0169 (5)	
C5A	0.5038 (4)	0.90923 (6)	0.33111 (16)	0.0175 (5)	
H5AA	0.4012	0.9019	0.2764	0.021*	
C6A	0.5780 (4)	0.88679 (6)	0.40110 (15)	0.0175 (5)	
H6AA	0.5296	0.8635	0.3936	0.021*	
C7A	0.7690 (4)	0.82152 (6)	0.60742 (15)	0.0163 (5)	
C8A	0.6995 (4)	0.78476 (6)	0.58883 (15)	0.0160 (5)	
C9A	0.5443 (4)	0.77613 (6)	0.50711 (15)	0.0170 (5)	
H9AA	0.4835	0.7934	0.4633	0.020*	
C10A	0.4817 (4)	0.74167 (6)	0.49183 (16)	0.0193 (5)	
C11A	0.5691 (4)	0.71538 (6)	0.55242 (17)	0.0220 (5)	
H11A	0.5253	0.6919	0.5391	0.026*	
C12A	0.7215 (4)	0.72408 (7)	0.63267 (17)	0.0219 (5)	
H12A	0.7839	0.7065	0.6754	0.026*	
C13A	0.7846 (4)	0.75853 (6)	0.65159 (16)	0.0189 (5)	
H13A	0.8868	0.7643	0.7079	0.023*	
C14A	0.8828 (4)	0.83316 (7)	0.70094 (17)	0.0272 (6)	
H14A	0.8557	0.8579	0.7058	0.041*	
H14B	1.0276	0.8298	0.7191	0.041*	
H14C	0.8396	0.8194	0.7390	0.041*	
O1B	1.0090 (3)	0.91068 (5)	1.14668 (12)	0.0260 (4)	
O2B	0.8666 (3)	0.86026 (4)	1.10339 (12)	0.0266 (4)	
O3B	0.4725 (4)	0.83754 (6)	0.80216 (16)	0.0654 (9)	
O4B	0.2945 (3)	0.87838 (5)	0.71768 (12)	0.0334 (5)	
O5B	0.3878 (3)	1.09140 (5)	0.83376 (11)	0.0236 (4)	
O6B	0.5168 (3)	1.14326 (5)	0.84719 (12)	0.0309 (5)	
N1B	0.8365 (3)	0.96614 (5)	1.05174 (13)	0.0190 (4)	
H1BA	0.9170	0.9595	1.1054	0.023*	
N2B	0.8059 (3)	1.00095 (5)	1.03058 (13)	0.0191 (4)	
N3B	0.8860 (3)	0.89130 (5)	1.09028 (14)	0.0198 (4)	
N4B	0.4247 (3)	0.86807 (6)	0.78739 (15)	0.0265 (5)	
N5B	0.5170 (3)	1.11357 (6)	0.87446 (14)	0.0212 (5)	
C1B	0.7396 (4)	0.94219 (6)	0.98780 (16)	0.0178 (5)	
C2B	0.7587 (4)	0.90569 (6)	1.00424 (15)	0.0167 (5)	
C3B	0.6541 (4)	0.88183 (6)	0.93861 (16)	0.0190 (5)	
H3BA	0.6687	0.8575	0.9506	0.023*	
C4B	0.5301 (4)	0.89357 (6)	0.85670 (16)	0.0192 (5)	
C5B	0.5034 (4)	0.92931 (7)	0.83717 (16)	0.0193 (5)	
H5BA	0.4142	0.9370	0.7800	0.023*	
C6B	0.6080 (4)	0.95299 (6)	0.90184 (15)	0.0187 (5)	
H6BA	0.5918	0.9772	0.8886	0.022*	
C7B	0.8768 (4)	1.02291 (6)	1.09502 (16)	0.0168 (5)	
C8B	0.8458 (4)	1.06007 (6)	1.06806 (15)	0.0164 (5)	
C9B	0.6992 (4)	1.06913 (6)	0.98502 (15)	0.0170 (5)	
H9BA	0.6163	1.0518	0.9459	0.020*	
C10B	0.6771 (4)	1.10396 (6)	0.96097 (15)	0.0168 (5)	
C11B	0.7940 (4)	1.13023 (6)	1.01474 (16)	0.0195 (5)	
H11B	0.7764	1.1538	0.9957	0.023*	
C12B	0.9374 (4)	1.12102 (6)	1.09710 (16)	0.0199 (5)	
H12B	1.0197	1.1385	1.1356	0.024*	
C13B	0.9629 (4)	1.08631 (6)	1.12452 (16)	0.0187 (5)	
H13B	1.0602	1.0805	1.1818	0.022*	
C14B	0.9801 (4)	1.01340 (7)	1.19055 (16)	0.0264 (6)	
H14D	0.9211	0.9919	1.2008	0.040*	
H14E	0.9627	1.0323	1.2260	0.040*	
H14F	1.1241	1.0098	1.2072	0.040*	
O1C	−0.1689 (3)	0.74191 (5)	0.38978 (12)	0.0275 (4)	
O2C	−0.1244 (3)	0.69066 (5)	0.44882 (12)	0.0296 (5)	
O3C	0.2722 (3)	0.67220 (5)	0.75401 (12)	0.0282 (4)	
O4C	0.5339 (3)	0.70556 (5)	0.81641 (12)	0.0257 (4)	
O5C	0.5986 (3)	0.91628 (5)	0.66988 (12)	0.0294 (4)	
O6C	0.5778 (3)	0.96939 (5)	0.62208 (14)	0.0362 (5)	
N1C	0.0454 (3)	0.79484 (5)	0.47944 (13)	0.0174 (4)	
H1CA	−0.0484	0.7885	0.4280	0.021*	
N2C	0.1181 (3)	0.82847 (5)	0.49206 (13)	0.0170 (4)	
N3C	−0.0874 (3)	0.72206 (5)	0.45348 (14)	0.0199 (4)	
N4C	0.3685 (3)	0.69869 (5)	0.75661 (13)	0.0192 (4)	
N5C	0.5215 (3)	0.93881 (6)	0.61320 (14)	0.0225 (5)	
C1C	0.1188 (4)	0.77154 (6)	0.54654 (16)	0.0163 (5)	
C2C	0.0576 (4)	0.73607 (6)	0.53636 (15)	0.0168 (5)	
C3C	0.1358 (4)	0.71267 (6)	0.60596 (16)	0.0175 (5)	
H3CA	0.0887	0.6893	0.5990	0.021*	
C4C	0.2817 (4)	0.72381 (6)	0.68475 (16)	0.0177 (5)	
C5C	0.3507 (4)	0.75844 (6)	0.69789 (16)	0.0183 (5)	
H5CA	0.4538	0.7655	0.7528	0.022*	
C6C	0.2673 (4)	0.78189 (6)	0.63043 (16)	0.0186 (5)	
H6CA	0.3094	0.8055	0.6399	0.022*	
C7C	0.0635 (4)	0.84757 (6)	0.42229 (16)	0.0155 (5)	
C8C	0.1381 (3)	0.88409 (6)	0.43691 (15)	0.0159 (5)	
C9C	0.2878 (3)	0.89426 (6)	0.51852 (15)	0.0166 (5)	
H9CA	0.3415	0.8782	0.5658	0.020*	
C10C	0.3557 (4)	0.92842 (7)	0.52864 (16)	0.0188 (5)	
C11C	0.2798 (4)	0.95319 (7)	0.46272 (17)	0.0213 (5)	
H11C	0.3289	0.9765	0.4722	0.026*	
C12C	0.1305 (4)	0.94299 (7)	0.38283 (17)	0.0224 (5)	
H12C	0.0747	0.9595	0.3366	0.027*	
C13C	0.0606 (4)	0.90885 (6)	0.36922 (16)	0.0184 (5)	
H13C	−0.0407	0.9022	0.3135	0.022*	
C14C	−0.0651 (4)	0.83538 (7)	0.33029 (16)	0.0213 (5)	
H14G	−0.0393	0.8106	0.3251	0.032*	
H14H	−0.0323	0.8489	0.2886	0.032*	
H14J	−0.2076	0.8387	0.3172	0.032*	

### Atomic displacement parameters (Å^2^)

**Table:**

	*U*^11^	*U*^22^	*U*^33^	*U*^12^	*U*^13^	*U*^23^
O1A	0.0358 (11)	0.0155 (9)	0.0182 (9)	−0.0019 (8)	0.0009 (9)	0.0017 (7)
O2A	0.0367 (11)	0.0116 (9)	0.0281 (10)	−0.0104 (8)	0.0075 (9)	−0.0028 (8)
O3A	0.0391 (12)	0.0254 (11)	0.0337 (11)	−0.0106 (9)	0.0063 (10)	0.0128 (9)
O4A	0.0245 (10)	0.0247 (10)	0.0195 (9)	0.0024 (8)	0.0022 (8)	0.0006 (8)
O5A	0.0274 (10)	0.0230 (10)	0.0221 (9)	−0.0029 (8)	0.0048 (8)	0.0017 (8)
O6A	0.0701 (17)	0.0199 (11)	0.0339 (12)	−0.0224 (11)	0.0051 (12)	−0.0043 (9)
N1A	0.0234 (11)	0.0084 (10)	0.0156 (10)	−0.0035 (8)	0.0041 (9)	−0.0013 (8)
N2A	0.0208 (11)	0.0089 (10)	0.0200 (10)	−0.0020 (8)	0.0072 (9)	−0.0017 (8)
N3A	0.0182 (10)	0.0132 (11)	0.0187 (10)	−0.0030 (8)	0.0051 (9)	−0.0032 (8)
N4A	0.0231 (11)	0.0165 (11)	0.0199 (11)	0.0023 (9)	0.0062 (10)	0.0015 (9)
N5A	0.0299 (13)	0.0203 (12)	0.0221 (11)	−0.0089 (10)	0.0111 (10)	−0.0029 (9)
C1A	0.0174 (12)	0.0113 (11)	0.0168 (11)	0.0008 (9)	0.0074 (10)	−0.0015 (9)
C2A	0.0149 (11)	0.0123 (12)	0.0156 (11)	−0.0025 (9)	0.0044 (10)	−0.0023 (9)
C3A	0.0190 (12)	0.0093 (11)	0.0226 (12)	−0.0013 (9)	0.0094 (11)	−0.0002 (9)
C4A	0.0187 (12)	0.0148 (12)	0.0176 (11)	0.0028 (9)	0.0081 (10)	0.0034 (9)
C5A	0.0172 (12)	0.0167 (13)	0.0181 (12)	−0.0025 (10)	0.0070 (10)	−0.0031 (9)
C6A	0.0190 (12)	0.0130 (12)	0.0196 (12)	−0.0033 (9)	0.0075 (10)	−0.0045 (9)
C7A	0.0178 (12)	0.0122 (12)	0.0178 (11)	−0.0015 (9)	0.0063 (10)	−0.0006 (9)
C8A	0.0184 (12)	0.0119 (12)	0.0185 (12)	0.0003 (9)	0.0086 (10)	0.0009 (9)
C9A	0.0212 (12)	0.0123 (12)	0.0174 (11)	−0.0008 (9)	0.0082 (10)	−0.0003 (9)
C10A	0.0226 (13)	0.0166 (13)	0.0190 (12)	−0.0051 (10)	0.0093 (11)	−0.0041 (10)
C11A	0.0289 (14)	0.0109 (12)	0.0270 (14)	−0.0014 (10)	0.0128 (12)	0.0016 (10)
C12A	0.0244 (13)	0.0147 (13)	0.0249 (13)	0.0023 (10)	0.0088 (12)	0.0080 (10)
C13A	0.0181 (12)	0.0168 (13)	0.0194 (12)	0.0001 (10)	0.0056 (10)	0.0015 (10)
C14A	0.0379 (16)	0.0167 (14)	0.0196 (13)	−0.0032 (12)	0.0052 (12)	−0.0002 (10)
O1B	0.0257 (10)	0.0190 (10)	0.0227 (9)	−0.0029 (8)	0.0000 (8)	0.0006 (8)
O2B	0.0398 (12)	0.0102 (9)	0.0266 (10)	0.0045 (8)	0.0110 (9)	0.0042 (7)
O3B	0.0775 (19)	0.0186 (12)	0.0493 (15)	0.0105 (12)	−0.0220 (14)	−0.0143 (11)
O4B	0.0345 (12)	0.0299 (12)	0.0223 (10)	−0.0046 (9)	−0.0010 (9)	−0.0023 (8)
O5B	0.0237 (10)	0.0202 (10)	0.0220 (9)	0.0008 (8)	0.0050 (8)	−0.0036 (8)
O6B	0.0378 (12)	0.0190 (10)	0.0285 (10)	0.0022 (9)	0.0070 (9)	0.0090 (8)
N1B	0.0247 (11)	0.0118 (10)	0.0159 (10)	−0.0002 (8)	0.0042 (9)	0.0012 (8)
N2B	0.0228 (11)	0.0123 (10)	0.0199 (10)	0.0007 (8)	0.0070 (9)	0.0012 (8)
N3B	0.0234 (11)	0.0143 (11)	0.0212 (11)	0.0017 (9)	0.0090 (10)	0.0008 (8)
N4B	0.0267 (12)	0.0207 (13)	0.0248 (12)	−0.0014 (10)	0.0039 (10)	−0.0042 (9)
N5B	0.0248 (11)	0.0169 (11)	0.0202 (11)	0.0031 (9)	0.0078 (10)	−0.0008 (9)
C1B	0.0202 (12)	0.0146 (12)	0.0194 (12)	−0.0002 (10)	0.0092 (10)	−0.0001 (10)
C2B	0.0179 (12)	0.0140 (12)	0.0175 (11)	0.0010 (9)	0.0068 (10)	0.0008 (9)
C3B	0.0201 (13)	0.0119 (12)	0.0236 (13)	−0.0002 (10)	0.0079 (11)	−0.0020 (10)
C4B	0.0185 (12)	0.0169 (13)	0.0204 (12)	−0.0024 (10)	0.0063 (11)	−0.0058 (10)
C5B	0.0193 (12)	0.0209 (13)	0.0169 (12)	0.0023 (10)	0.0069 (10)	0.0022 (10)
C6B	0.0217 (13)	0.0153 (13)	0.0178 (12)	0.0034 (10)	0.0071 (11)	0.0026 (9)
C7B	0.0205 (12)	0.0113 (12)	0.0181 (12)	0.0010 (9)	0.0076 (10)	0.0001 (9)
C8B	0.0191 (12)	0.0130 (12)	0.0165 (11)	−0.0008 (9)	0.0071 (10)	−0.0017 (9)
C9B	0.0177 (12)	0.0154 (12)	0.0172 (11)	−0.0006 (9)	0.0068 (10)	−0.0022 (9)
C10B	0.0186 (12)	0.0155 (12)	0.0149 (11)	0.0038 (9)	0.0057 (10)	0.0025 (9)
C11B	0.0247 (13)	0.0114 (12)	0.0228 (13)	0.0010 (10)	0.0103 (11)	0.0001 (10)
C12B	0.0235 (13)	0.0139 (12)	0.0207 (12)	−0.0048 (10)	0.0079 (11)	−0.0062 (10)
C13B	0.0194 (12)	0.0169 (13)	0.0169 (11)	−0.0005 (10)	0.0050 (10)	−0.0020 (9)
C14B	0.0379 (16)	0.0157 (13)	0.0198 (13)	0.0033 (12)	0.0067 (12)	−0.0006 (10)
O1C	0.0319 (11)	0.0156 (10)	0.0234 (10)	−0.0011 (8)	0.0004 (9)	0.0041 (8)
O2C	0.0362 (11)	0.0106 (9)	0.0299 (10)	−0.0063 (8)	0.0024 (9)	−0.0026 (8)
O3C	0.0300 (11)	0.0185 (10)	0.0316 (11)	−0.0023 (8)	0.0088 (9)	0.0074 (8)
O4C	0.0213 (10)	0.0264 (11)	0.0211 (9)	0.0031 (8)	0.0010 (8)	0.0028 (8)
O5C	0.0267 (10)	0.0303 (11)	0.0234 (10)	−0.0038 (9)	0.0032 (9)	−0.0006 (8)
O6C	0.0383 (12)	0.0194 (11)	0.0402 (12)	−0.0093 (9)	0.0064 (10)	−0.0093 (9)
N1C	0.0215 (11)	0.0100 (10)	0.0162 (10)	−0.0016 (8)	0.0035 (9)	−0.0005 (8)
N2C	0.0199 (11)	0.0096 (10)	0.0212 (10)	−0.0013 (8)	0.0084 (9)	−0.0019 (8)
N3C	0.0195 (11)	0.0153 (11)	0.0211 (11)	−0.0016 (9)	0.0050 (9)	−0.0014 (9)
N4C	0.0214 (11)	0.0160 (11)	0.0179 (10)	0.0026 (9)	0.0061 (9)	0.0019 (8)
N5C	0.0213 (11)	0.0214 (12)	0.0235 (11)	−0.0028 (9)	0.0084 (10)	−0.0058 (9)
C1C	0.0197 (12)	0.0114 (12)	0.0190 (12)	0.0000 (9)	0.0096 (10)	−0.0004 (9)
C2C	0.0188 (12)	0.0109 (12)	0.0170 (11)	−0.0006 (9)	0.0041 (10)	−0.0007 (9)
C3C	0.0203 (12)	0.0099 (12)	0.0222 (12)	−0.0004 (9)	0.0090 (11)	−0.0013 (9)
C4C	0.0185 (12)	0.0155 (12)	0.0185 (12)	0.0026 (10)	0.0073 (10)	0.0039 (9)
C5C	0.0201 (12)	0.0158 (13)	0.0178 (12)	−0.0025 (10)	0.0068 (10)	−0.0012 (9)
C6C	0.0227 (13)	0.0128 (12)	0.0197 (12)	−0.0041 (10)	0.0084 (11)	−0.0022 (9)
C7C	0.0156 (11)	0.0097 (11)	0.0197 (12)	−0.0006 (9)	0.0061 (10)	−0.0005 (9)
C8C	0.0163 (12)	0.0143 (12)	0.0176 (11)	0.0007 (9)	0.0078 (10)	0.0007 (9)
C9C	0.0170 (12)	0.0139 (12)	0.0165 (11)	0.0021 (9)	0.0048 (10)	0.0011 (9)
C10C	0.0157 (12)	0.0184 (13)	0.0208 (12)	−0.0013 (10)	0.0063 (10)	−0.0045 (10)
C11C	0.0231 (13)	0.0113 (12)	0.0317 (14)	−0.0014 (10)	0.0137 (12)	−0.0002 (10)
C12C	0.0244 (13)	0.0183 (13)	0.0239 (13)	0.0039 (11)	0.0097 (11)	0.0073 (10)
C13C	0.0186 (12)	0.0163 (13)	0.0184 (12)	0.0005 (10)	0.0061 (10)	0.0007 (10)
C14C	0.0243 (13)	0.0148 (13)	0.0183 (12)	−0.0034 (10)	0.0027 (11)	−0.0001 (10)

### Geometric parameters (Å, º)

**Table:**

O1A—N3A	1.240 (3)	C5B—C6B	1.373 (3)
O2A—N3A	1.229 (3)	C5B—H5BA	0.9500
O3A—N4A	1.231 (3)	C6B—H6BA	0.9500
O4A—N4A	1.234 (3)	C7B—C8B	1.483 (3)
O5A—N5A	1.226 (3)	C7B—C14B	1.500 (3)
O6A—N5A	1.222 (3)	C8B—C9B	1.396 (3)
N1A—C1A	1.360 (3)	C8B—C13B	1.399 (3)
N1A—N2A	1.375 (3)	C9B—C10B	1.385 (3)
N1A—H1AA	0.8800	C9B—H9BA	0.9500
N2A—C7A	1.291 (3)	C10B—C11B	1.381 (3)
N3A—C2A	1.450 (3)	C11B—C12B	1.381 (4)
N4A—C4A	1.459 (3)	C11B—H11B	0.9500
N5A—C10A	1.476 (3)	C12B—C13B	1.395 (3)
C1A—C6A	1.419 (3)	C12B—H12B	0.9500
C1A—C2A	1.420 (3)	C13B—H13B	0.9500
C2A—C3A	1.392 (3)	C14B—H14D	0.9800
C3A—C4A	1.370 (3)	C14B—H14E	0.9800
C3A—H3AA	0.9500	C14B—H14F	0.9800
C4A—C5A	1.402 (3)	O1C—N3C	1.240 (3)
C5A—C6A	1.370 (3)	O2C—N3C	1.230 (3)
C5A—H5AA	0.9500	O3C—N4C	1.228 (3)
C6A—H6AA	0.9500	O4C—N4C	1.230 (3)
C7A—C8A	1.486 (3)	O5C—N5C	1.231 (3)
C7A—C14A	1.500 (3)	O6C—N5C	1.231 (3)
C8A—C13A	1.398 (3)	N1C—C1C	1.358 (3)
C8A—C9A	1.400 (3)	N1C—N2C	1.377 (3)
C9A—C10A	1.387 (3)	N1C—H1CA	0.8800
C9A—H9AA	0.9500	N2C—C7C	1.293 (3)
C10A—C11A	1.382 (3)	N3C—C2C	1.449 (3)
C11A—C12A	1.377 (4)	N4C—C4C	1.462 (3)
C11A—H11A	0.9500	N5C—C10C	1.479 (3)
C12A—C13A	1.391 (3)	C1C—C2C	1.420 (3)
C12A—H12A	0.9500	C1C—C6C	1.423 (3)
C13A—H13A	0.9500	C2C—C3C	1.390 (3)
C14A—H14A	0.9800	C3C—C4C	1.369 (3)
C14A—H14B	0.9800	C3C—H3CA	0.9500
C14A—H14C	0.9800	C4C—C5C	1.405 (3)
O1B—N3B	1.240 (3)	C5C—C6C	1.370 (3)
O2B—N3B	1.230 (3)	C5C—H5CA	0.9500
O3B—N4B	1.217 (3)	C6C—H6CA	0.9500
O4B—N4B	1.220 (3)	C7C—C8C	1.486 (3)
O5B—N5B	1.239 (3)	C7C—C14C	1.503 (3)
O6B—N5B	1.227 (3)	C8C—C9C	1.399 (3)
N1B—C1B	1.362 (3)	C8C—C13C	1.403 (3)
N1B—N2B	1.375 (3)	C9C—C10C	1.386 (3)
N1B—H1BA	0.8800	C9C—H9CA	0.9500
N2B—C7B	1.294 (3)	C10C—C11C	1.384 (4)
N3B—C2B	1.456 (3)	C11C—C12C	1.380 (4)
N4B—C4B	1.464 (3)	C11C—H11C	0.9500
N5B—C10B	1.475 (3)	C12C—C13C	1.389 (3)
C1B—C6B	1.415 (3)	C12C—H12C	0.9500
C1B—C2B	1.423 (3)	C13C—H13C	0.9500
C2B—C3B	1.386 (3)	C14C—H14G	0.9800
C3B—C4B	1.364 (3)	C14C—H14H	0.9800
C3B—H3BA	0.9500	C14C—H14J	0.9800
C4B—C5B	1.404 (3)		
			
C1A—N1A—N2A	118.9 (2)	C5B—C6B—H6BA	119.2
C1A—N1A—H1AA	120.5	C1B—C6B—H6BA	119.2
N2A—N1A—H1AA	120.5	N2B—C7B—C8B	114.5 (2)
C7A—N2A—N1A	117.0 (2)	N2B—C7B—C14B	125.3 (2)
O2A—N3A—O1A	122.1 (2)	C8B—C7B—C14B	120.2 (2)
O2A—N3A—C2A	118.8 (2)	C9B—C8B—C13B	119.2 (2)
O1A—N3A—C2A	119.1 (2)	C9B—C8B—C7B	119.8 (2)
O3A—N4A—O4A	123.7 (2)	C13B—C8B—C7B	120.9 (2)
O3A—N4A—C4A	118.4 (2)	C10B—C9B—C8B	118.5 (2)
O4A—N4A—C4A	117.9 (2)	C10B—C9B—H9BA	120.8
O6A—N5A—O5A	123.2 (2)	C8B—C9B—H9BA	120.8
O6A—N5A—C10A	118.1 (2)	C11B—C10B—C9B	123.4 (2)
O5A—N5A—C10A	118.7 (2)	C11B—C10B—N5B	118.3 (2)
N1A—C1A—C6A	120.0 (2)	C9B—C10B—N5B	118.3 (2)
N1A—C1A—C2A	123.1 (2)	C10B—C11B—C12B	117.7 (2)
C6A—C1A—C2A	116.9 (2)	C10B—C11B—H11B	121.1
C3A—C2A—C1A	121.7 (2)	C12B—C11B—H11B	121.1
C3A—C2A—N3A	116.2 (2)	C11B—C12B—C13B	120.9 (2)
C1A—C2A—N3A	122.1 (2)	C11B—C12B—H12B	119.6
C4A—C3A—C2A	118.7 (2)	C13B—C12B—H12B	119.6
C4A—C3A—H3AA	120.7	C12B—C13B—C8B	120.3 (2)
C2A—C3A—H3AA	120.7	C12B—C13B—H13B	119.8
C3A—C4A—C5A	121.9 (2)	C8B—C13B—H13B	119.8
C3A—C4A—N4A	118.5 (2)	C7B—C14B—H14D	109.5
C5A—C4A—N4A	119.5 (2)	C7B—C14B—H14E	109.5
C6A—C5A—C4A	119.2 (2)	H14D—C14B—H14E	109.5
C6A—C5A—H5AA	120.4	C7B—C14B—H14F	109.5
C4A—C5A—H5AA	120.4	H14D—C14B—H14F	109.5
C5A—C6A—C1A	121.5 (2)	H14E—C14B—H14F	109.5
C5A—C6A—H6AA	119.2	C1C—N1C—N2C	119.8 (2)
C1A—C6A—H6AA	119.2	C1C—N1C—H1CA	120.1
N2A—C7A—C8A	115.2 (2)	N2C—N1C—H1CA	120.1
N2A—C7A—C14A	125.4 (2)	C7C—N2C—N1C	116.2 (2)
C8A—C7A—C14A	119.4 (2)	O2C—N3C—O1C	122.3 (2)
C13A—C8A—C9A	119.0 (2)	O2C—N3C—C2C	118.5 (2)
C13A—C8A—C7A	121.4 (2)	O1C—N3C—C2C	119.3 (2)
C9A—C8A—C7A	119.6 (2)	O3C—N4C—O4C	124.0 (2)
C10A—C9A—C8A	118.3 (2)	O3C—N4C—C4C	118.3 (2)
C10A—C9A—H9AA	120.9	O4C—N4C—C4C	117.7 (2)
C8A—C9A—H9AA	120.9	O5C—N5C—O6C	123.7 (2)
C11A—C10A—C9A	123.1 (2)	O5C—N5C—C10C	118.3 (2)
C11A—C10A—N5A	118.7 (2)	O6C—N5C—C10C	118.0 (2)
C9A—C10A—N5A	118.2 (2)	N1C—C1C—C2C	122.5 (2)
C12A—C11A—C10A	118.3 (2)	N1C—C1C—C6C	120.3 (2)
C12A—C11A—H11A	120.9	C2C—C1C—C6C	117.1 (2)
C10A—C11A—H11A	120.9	C3C—C2C—C1C	121.4 (2)
C11A—C12A—C13A	120.4 (2)	C3C—C2C—N3C	116.2 (2)
C11A—C12A—H12A	119.8	C1C—C2C—N3C	122.4 (2)
C13A—C12A—H12A	119.8	C4C—C3C—C2C	119.0 (2)
C12A—C13A—C8A	120.9 (2)	C4C—C3C—H3CA	120.5
C12A—C13A—H13A	119.6	C2C—C3C—H3CA	120.5
C8A—C13A—H13A	119.6	C3C—C4C—C5C	121.9 (2)
C7A—C14A—H14A	109.5	C3C—C4C—N4C	118.7 (2)
C7A—C14A—H14B	109.5	C5C—C4C—N4C	119.5 (2)
H14A—C14A—H14B	109.5	C6C—C5C—C4C	119.1 (2)
C7A—C14A—H14C	109.5	C6C—C5C—H5CA	120.5
H14A—C14A—H14C	109.5	C4C—C5C—H5CA	120.5
H14B—C14A—H14C	109.5	C5C—C6C—C1C	121.4 (2)
C1B—N1B—N2B	118.5 (2)	C5C—C6C—H6CA	119.3
C1B—N1B—H1BA	120.7	C1C—C6C—H6CA	119.3
N2B—N1B—H1BA	120.7	N2C—C7C—C8C	115.7 (2)
C7B—N2B—N1B	116.9 (2)	N2C—C7C—C14C	125.0 (2)
O2B—N3B—O1B	123.1 (2)	C8C—C7C—C14C	119.3 (2)
O2B—N3B—C2B	118.1 (2)	C9C—C8C—C13C	119.0 (2)
O1B—N3B—C2B	118.9 (2)	C9C—C8C—C7C	120.3 (2)
O3B—N4B—O4B	123.2 (2)	C13C—C8C—C7C	120.7 (2)
O3B—N4B—C4B	118.1 (2)	C10C—C9C—C8C	118.3 (2)
O4B—N4B—C4B	118.7 (2)	C10C—C9C—H9CA	120.8
O6B—N5B—O5B	123.4 (2)	C8C—C9C—H9CA	120.8
O6B—N5B—C10B	118.3 (2)	C11C—C10C—C9C	123.3 (2)
O5B—N5B—C10B	118.3 (2)	C11C—C10C—N5C	118.1 (2)
N1B—C1B—C6B	120.6 (2)	C9C—C10C—N5C	118.6 (2)
N1B—C1B—C2B	122.4 (2)	C12C—C11C—C10C	117.8 (2)
C6B—C1B—C2B	117.0 (2)	C12C—C11C—H11C	121.1
C3B—C2B—C1B	121.3 (2)	C10C—C11C—H11C	121.1
C3B—C2B—N3B	116.4 (2)	C11C—C12C—C13C	120.8 (2)
C1B—C2B—N3B	122.3 (2)	C11C—C12C—H12C	119.6
C4B—C3B—C2B	119.4 (2)	C13C—C12C—H12C	119.6
C4B—C3B—H3BA	120.3	C12C—C13C—C8C	120.7 (2)
C2B—C3B—H3BA	120.3	C12C—C13C—H13C	119.6
C3B—C4B—C5B	121.7 (2)	C8C—C13C—H13C	119.6
C3B—C4B—N4B	118.8 (2)	C7C—C14C—H14G	109.5
C5B—C4B—N4B	119.5 (2)	C7C—C14C—H14H	109.5
C6B—C5B—C4B	119.0 (2)	H14G—C14C—H14H	109.5
C6B—C5B—H5BA	120.5	C7C—C14C—H14J	109.5
C4B—C5B—H5BA	120.5	H14G—C14C—H14J	109.5
C5B—C6B—C1B	121.5 (2)	H14H—C14C—H14J	109.5
			
C1A—N1A—N2A—C7A	−171.6 (2)	N1B—C1B—C6B—C5B	−177.7 (2)
N2A—N1A—C1A—C6A	4.3 (3)	C2B—C1B—C6B—C5B	0.0 (3)
N2A—N1A—C1A—C2A	−177.5 (2)	N1B—N2B—C7B—C8B	177.9 (2)
N1A—C1A—C2A—C3A	179.3 (2)	N1B—N2B—C7B—C14B	−3.1 (4)
C6A—C1A—C2A—C3A	−2.5 (3)	N2B—C7B—C8B—C9B	19.2 (3)
N1A—C1A—C2A—N3A	−1.2 (3)	C14B—C7B—C8B—C9B	−159.9 (2)
C6A—C1A—C2A—N3A	177.0 (2)	N2B—C7B—C8B—C13B	−160.4 (2)
O2A—N3A—C2A—C3A	0.1 (3)	C14B—C7B—C8B—C13B	20.6 (3)
O1A—N3A—C2A—C3A	178.9 (2)	C13B—C8B—C9B—C10B	1.0 (3)
O2A—N3A—C2A—C1A	−179.4 (2)	C7B—C8B—C9B—C10B	−178.5 (2)
O1A—N3A—C2A—C1A	−0.6 (3)	C8B—C9B—C10B—C11B	0.5 (4)
C1A—C2A—C3A—C4A	2.8 (3)	C8B—C9B—C10B—N5B	−177.8 (2)
N3A—C2A—C3A—C4A	−176.7 (2)	O6B—N5B—C10B—C11B	12.8 (3)
C2A—C3A—C4A—C5A	−0.4 (4)	O5B—N5B—C10B—C11B	−166.1 (2)
C2A—C3A—C4A—N4A	179.9 (2)	O6B—N5B—C10B—C9B	−168.8 (2)
O3A—N4A—C4A—C3A	19.8 (3)	O5B—N5B—C10B—C9B	12.3 (3)
O4A—N4A—C4A—C3A	−160.9 (2)	C9B—C10B—C11B—C12B	−1.1 (4)
O3A—N4A—C4A—C5A	−159.9 (2)	N5B—C10B—C11B—C12B	177.2 (2)
O4A—N4A—C4A—C5A	19.4 (3)	C10B—C11B—C12B—C13B	0.2 (4)
C3A—C4A—C5A—C6A	−2.3 (4)	C11B—C12B—C13B—C8B	1.3 (4)
N4A—C4A—C5A—C6A	177.4 (2)	C9B—C8B—C13B—C12B	−1.9 (3)
C4A—C5A—C6A—C1A	2.6 (4)	C7B—C8B—C13B—C12B	177.6 (2)
N1A—C1A—C6A—C5A	178.0 (2)	C1C—N1C—N2C—C7C	170.1 (2)
C2A—C1A—C6A—C5A	−0.2 (3)	N2C—N1C—C1C—C2C	−176.7 (2)
N1A—N2A—C7A—C8A	−177.61 (19)	N2C—N1C—C1C—C6C	1.4 (3)
N1A—N2A—C7A—C14A	2.6 (4)	N1C—C1C—C2C—C3C	179.7 (2)
N2A—C7A—C8A—C13A	161.1 (2)	C6C—C1C—C2C—C3C	1.5 (3)
C14A—C7A—C8A—C13A	−19.1 (3)	N1C—C1C—C2C—N3C	0.2 (4)
N2A—C7A—C8A—C9A	−19.5 (3)	C6C—C1C—C2C—N3C	−178.0 (2)
C14A—C7A—C8A—C9A	160.3 (2)	O2C—N3C—C2C—C3C	−3.3 (3)
C13A—C8A—C9A—C10A	−0.1 (3)	O1C—N3C—C2C—C3C	177.1 (2)
C7A—C8A—C9A—C10A	−179.6 (2)	O2C—N3C—C2C—C1C	176.2 (2)
C8A—C9A—C10A—C11A	−2.0 (4)	O1C—N3C—C2C—C1C	−3.4 (3)
C8A—C9A—C10A—N5A	176.1 (2)	C1C—C2C—C3C—C4C	−3.0 (4)
O6A—N5A—C10A—C11A	−1.2 (4)	N3C—C2C—C3C—C4C	176.5 (2)
O5A—N5A—C10A—C11A	176.2 (2)	C2C—C3C—C4C—C5C	1.7 (4)
O6A—N5A—C10A—C9A	−179.3 (2)	C2C—C3C—C4C—N4C	−177.7 (2)
O5A—N5A—C10A—C9A	−1.9 (3)	O3C—N4C—C4C—C3C	−21.0 (3)
C9A—C10A—C11A—C12A	2.0 (4)	O4C—N4C—C4C—C3C	159.4 (2)
N5A—C10A—C11A—C12A	−176.0 (2)	O3C—N4C—C4C—C5C	159.5 (2)
C10A—C11A—C12A—C13A	0.0 (4)	O4C—N4C—C4C—C5C	−20.1 (3)
C11A—C12A—C13A—C8A	−2.0 (4)	C3C—C4C—C5C—C6C	1.1 (4)
C9A—C8A—C13A—C12A	2.1 (4)	N4C—C4C—C5C—C6C	−179.4 (2)
C7A—C8A—C13A—C12A	−178.5 (2)	C4C—C5C—C6C—C1C	−2.7 (4)
C1B—N1B—N2B—C7B	170.5 (2)	N1C—C1C—C6C—C5C	−176.8 (2)
N2B—N1B—C1B—C6B	−0.9 (3)	C2C—C1C—C6C—C5C	1.4 (3)
N2B—N1B—C1B—C2B	−178.5 (2)	N1C—N2C—C7C—C8C	178.35 (19)
N1B—C1B—C2B—C3B	178.2 (2)	N1C—N2C—C7C—C14C	−2.1 (3)
C6B—C1B—C2B—C3B	0.5 (3)	N2C—C7C—C8C—C9C	11.8 (3)
N1B—C1B—C2B—N3B	−1.1 (4)	C14C—C7C—C8C—C9C	−167.8 (2)
C6B—C1B—C2B—N3B	−178.8 (2)	N2C—C7C—C8C—C13C	−168.6 (2)
O2B—N3B—C2B—C3B	−12.4 (3)	C14C—C7C—C8C—C13C	11.9 (3)
O1B—N3B—C2B—C3B	167.4 (2)	C13C—C8C—C9C—C10C	−1.0 (3)
O2B—N3B—C2B—C1B	167.0 (2)	C7C—C8C—C9C—C10C	178.7 (2)
O1B—N3B—C2B—C1B	−13.2 (3)	C8C—C9C—C10C—C11C	1.5 (4)
C1B—C2B—C3B—C4B	−0.1 (4)	C8C—C9C—C10C—N5C	−176.7 (2)
N3B—C2B—C3B—C4B	179.2 (2)	O5C—N5C—C10C—C11C	−175.2 (2)
C2B—C3B—C4B—C5B	−0.8 (4)	O6C—N5C—C10C—C11C	3.1 (3)
C2B—C3B—C4B—N4B	178.4 (2)	O5C—N5C—C10C—C9C	3.1 (3)
O3B—N4B—C4B—C3B	−9.8 (4)	O6C—N5C—C10C—C9C	−178.5 (2)
O4B—N4B—C4B—C3B	170.6 (2)	C9C—C10C—C11C—C12C	−0.7 (4)
O3B—N4B—C4B—C5B	169.4 (3)	N5C—C10C—C11C—C12C	177.5 (2)
O4B—N4B—C4B—C5B	−10.2 (4)	C10C—C11C—C12C—C13C	−0.6 (4)
C3B—C4B—C5B—C6B	1.3 (4)	C11C—C12C—C13C—C8C	1.1 (4)
N4B—C4B—C5B—C6B	−177.9 (2)	C9C—C8C—C13C—C12C	−0.3 (3)
C4B—C5B—C6B—C1B	−0.9 (4)	C7C—C8C—C13C—C12C	−179.9 (2)

### Hydrogen-bond geometry (Å, º)

**Table:**

*D*—H···*A*	*D*—H	H···*A*	*D*···*A*	*D*—H···*A*
N1*A*—H1*AA*···O1*A*	0.88	1.98	2.615 (3)	128
N1*B*—H1*BA*···O1*B*	0.88	2.01	2.637 (3)	127
N1*C*—H1*CA*···O1*C*	0.88	1.98	2.617 (3)	129
C3*A*—H3*AA*···O2*A*^i^	0.95	2.44	3.320 (3)	154
C3*B*—H3*BA*···O2*C*^ii^	0.95	2.40	3.188 (3)	140
C3*C*—H3*CA*···O2*B*^iii^	0.95	2.52	3.413 (3)	156
C14*B*—H14*F*···O4*A*^iv^	0.98	2.53	3.316 (4)	137
C14*C*—H14*J*···O4*C*^iii^	0.98	2.54	3.253 (4)	129

Symmetry codes: (i) −*x*+2, −*y*+2, −*z*+1; (ii) *x*+1, −*y*+3/2, *z*+1/2; (iii) *x*−1, −*y*+3/2, *z*−1/2; (iv) *x*+1, *y*, *z*+1.
